# An analysis of the global pharmacy workforce capacity trends from 2006 to 2012

**DOI:** 10.1186/s12960-018-0267-y

**Published:** 2018-01-11

**Authors:** Ian Bates, Christopher John, Priyanka Seegobin, Andreia Bruno

**Affiliations:** 1 FIP Collaborating Centre, UCL School of Pharmacy, 29-39 Brunswick Square, London, WC1N 1AX United Kingdom; 2 Royal Pharmaceutical Society, Global Pharmacy Workforce Observatory, 66-68 East Smithfield, London, E1W 1AW United Kingdom; 3 International Pharmaceutical Federation (FIP), 2517 JP The Hague, The Netherlands

**Keywords:** Pharmacy workforce, Global, Capacity, Healthcare, Workforce trends

## Abstract

**Background:**

Human resources for health are at a critical low. The World Health Organization estimates that the current shortage of health workers, including pharmacists, is in excess of 7.2 million worldwide and that, by 2035, the shortage will reach 12.9 million. Pharmacists, in particular, are lacking in the workforce in many countries. The International Pharmaceutical Federation (FIP) and academic partners have conducted periodic global pharmacy workforce surveys in 2006, 2009 and 2012 which have monitored and reported on the status of the pharmacy workforce at the country and territory levels. This current analysis is a synthesis of workforce capacity data from these date points to provide an overview of the global trends and changes to pharmacy workforce capacity over this time period.

**Methods:**

The methodology proceeded with accessing workforce capacity data collated in 2006, 2009 and 2012 held on file at the FIP Collaborating Centre. This data had previously been validated and made available to WHO Human Resources for Health. The data focused (due to limitations from 2006 databank) on pharmacist workforce capacity. Countries and territories were identified that had data available across at least two of the three time points (2006, 2009 and 2012). Missing time-point data for some countries (data gaps) were subject, where possible, to literature and online data searching to capture possible missing data. Country-level capacity data were plotted against time to identify trends coupled with comparative analysis of the trends.

**Results:**

The countries and territories identified as having valid data for each of the time points 2006, 2009 and 2012 were present in all WHO regions, with Europe having the most countries with data available and South East Asia the fewest.

All WHO regions have experienced an increase in the density of pharmacists (measured as number of pharmacists per 10 000 population) over the period 2006–2012. However, some countries show a reduction in the density of pharmacists. African countries show large relative increases in acceleration of capacity building but remain significantly behind in terms of absolute capacity per capita. South East Asian and Middle Eastern countries also show large proportional changes in pharmacist workforce.

**Conclusion:**

The global trend is an increase in workforce across all nations and regions, and this is a move in the right direction towards improved access to, and availability of, pharmaceutical expertise. However, there is still much to be done, with some regions and low-income countries still displaying a disproportionately low number of pharmacists on small overall capacity for delivering pharmacy services.

**Electronic supplementary material:**

The online version of this article (10.1186/s12960-018-0267-y) contains supplementary material, which is available to authorized users.

## Background

Human resources for health are at a critical low. The World Health Organization estimates that the current shortage of health workers, including pharmacists, is in excess of 7.2 million worldwide and that, by 2035, the shortage will reach over 13 million [1]. Pharmacists,^1^ in particular, are lacking in the workforce in many countries. In addition, education and training needs to be strengthened globally.

The International Pharmaceutical Federation (FIP) Global Pharmacy Workforce surveys conducted in 2006 [2], 2009 [3] and 2012 [4] analysed, monitored and reported on the status of the pharmacy workforce at the country and territory levels. In 2012, the survey collected workforce data for 90 countries and territories representing 2.5 million pharmacists and nearly one and a half million technicians and support workers. Fifty-six countries responded to the 2009 report and 34 to the 2006 survey.

A key message from the 2006, 2009 and 2012 Global Pharmacy Workforce reports was that pharmacy workforce density varied considerably between countries and WHO regions and generally correlated with population numbers and country-level economic development indicators. Those countries and territories with lower economic indicators tended to have relatively fewer pharmacists and pharmacy technicians. With the recent launch of global pharmaceutical workforce development goals (WDGs: http://www.fip.org/educationreports), FIP have highlighted that workforce intelligence is a critical factor in planning for transformative change; understanding the global trends in workforce capacity, in addition to the absolute capacity values, adds an important dimension to policy planning.

Access to high-quality health services is vital for the delivery of a nation’s positive health outcomes. For example, the reduction of morbidity associated with long-term conditions requires access to pharmacy teams who can provide medicines and services, to ensure their responsible use. Ensuring the availability of an appropriately skilled pharmacy workforce within services and facilities with effective distribution across a nation is an important approach for improving equitable access. Each country and territory in this report started from a different baseline in terms of the number of pharmacists. The impact of changes in the density of the pharmacy workforce (whether this is an increase or decrease) on health outcomes is difficult to judge. Additionally, changing epidemiology and disease burden at a country level as well as population increases need to be considered and an assessment made as to whether the development of pharmacy human resources has adapted and made an impact over time.

The proportion of women in the pharmacy workforce continues to increase resulting in more part-time working and therefore a greater headcount being required to meet demand. Productivity of pharmacists in many locations is being increased due to technology (use of robotics) and optimising skill mix (supported by changing scope of the pharmacy technician role) [5]. Conversely, demand for pharmacists is also increasing in some areas because of the creation of new roles in order to mitigate shortages in other healthcare professions such as medicine and nursing.

Policy developments from WHO Human Resources for Health clearly indicate current and projected chronic shortages of the healthcare workforce and the availability of a skilled pharmacy workforce for access to expertise and essential medicines has been made clear [6, 7]. The objective of this paper is to describe the key issues and current trends affecting the global pharmacy workforce by providing a synthesis of the changes to pharmacy workforce capacity over the time period covering 2006, 2009 and 2012. In particular, the data provides a focus on workforce distribution over time, to provide better intelligence on trends in capacity by country.

## Methods

The methodology proceeded with accessing workforce capacity data collated in 2006, 2009 and 2012 held on file at the FIP Collaborating Centre (*see references* [2–4]). The data focused (due to limitations from 2006 databank) on pharmacist workforce capacity. Countries and territories were identified that had data available across at least two of the three time points (2006, 2009 and 2012). Missing time-point data for some countries (data gaps) were subject, where possible, to literature and online data searching to capture possible missing data. Country-level capacity data were plotted against time to identify trends coupled with comparative analysis of the trends. Workforce capacity is measured as ‘density’ which is the number pharmacists per 10 000 population, and serves as a standardised measure for the purposes of this report.

The base workforce capacity data for years 2006, 2009 and 2012 were collated from repeat surveys of national agencies (professional leadership bodies, health workforce regulators, ministries) and conducted by the FIP Collaborating Centre, University College London, School of Pharmacy and the FIP Education directorate (FIP*Ed*). The collated data related to pharmacy workforce capacity and was available in English, French and Spanish. Surveys had been conducted using email contacts derived from FIP, website information with repeat follow-up for non-responders. This data had previously been validated and made available to WHO Human Resources for Health [8]. Gaps in the date-stamped data were identified and literature and web searches were conducted where possible to minimise data gaps. The aim was to have three valid, date-stamped data points per country; for a small minority of countries in this sample, only two date points were able to be used.

Additional country-level variables included national population (as reported by the World Bank for each date stamp), pharmacist registries, and national demographic data such as Gross National Income & Expenditure (World Bank data repositories). A full methodology on the FIP survey process and data validation has been previously reported [8].

A combined dataset of national workforce capacity was constructed that captured data at the three date points (2006, 2009, 2012 contiguous cases). This new dataset was cleaned and prepared for analysis using SPSS Statistics v22. Limitations include a reliance, for some data coordinates, on published data and secondary sources for national data (rather than drawn from the FIP Survey method). Quality assurance was based on previous reporting and identification of data outliers for cross-checking. A fuller methodology has been previously reported [8].

## Results

**Fig. 1:**
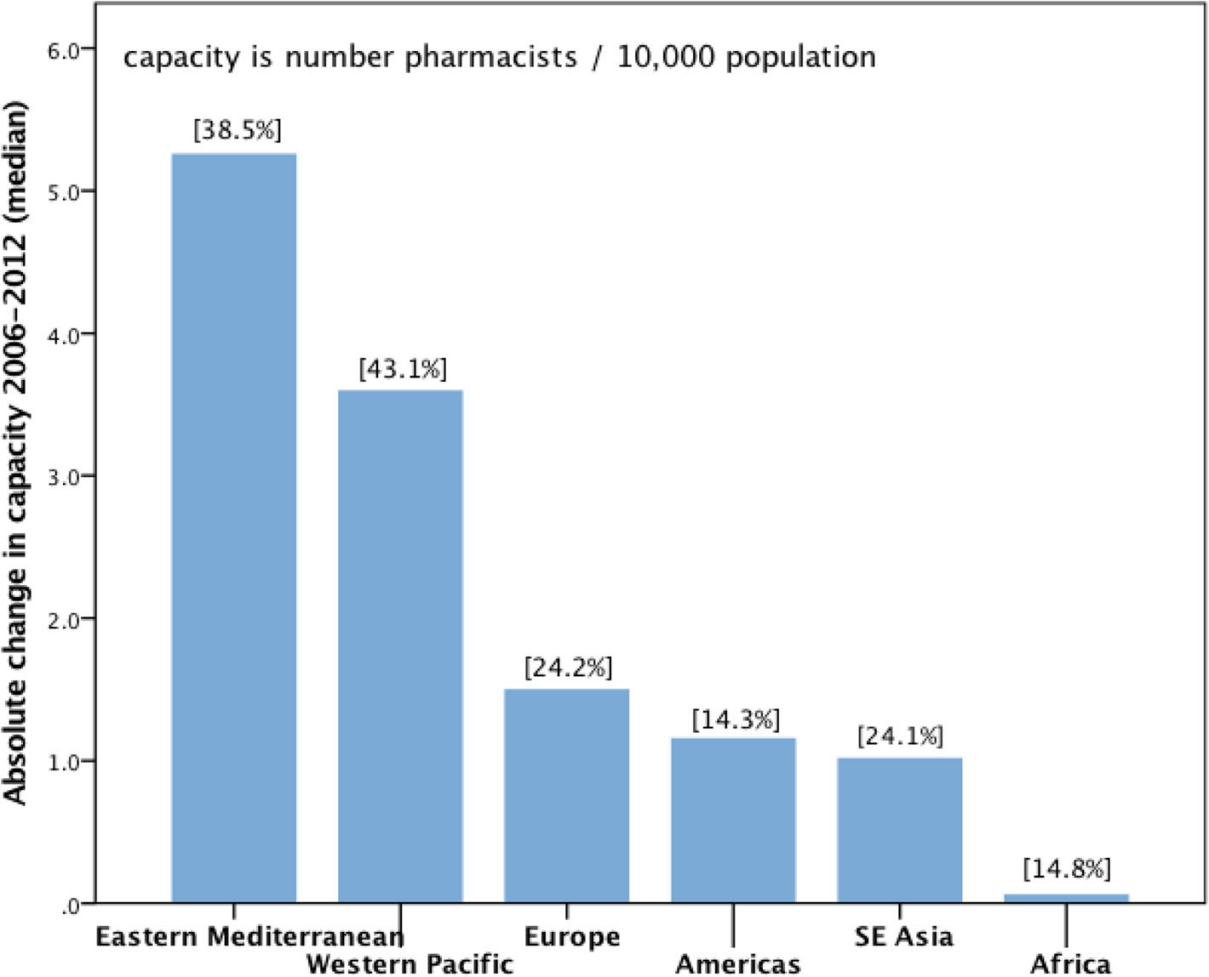
Change in absolute capacity by WHO region (units are number pharmacists/10 000 population) from 2006 to 2012. Percent figures in brackets indicate the mean proportional change in density from 2006 baseline (*N* = 50 countries)

Matching data across the three time points (2006, 2009, 2012) and charting pharmacist capacity across these dates provides an overview as seen in Fig. 1 compared across aggregated WHO regional groups of countries. Figure 1 illustrates both the mean percentage change in pharmacist density for each WHO region and also the important absolute change in pharmacist density per capita. The data comprises 58 countries with at least two date points (out of three) and 50 countries with all three date points (2006, 2009, 2012) in total representing around 30% of current WHO member states.

**Fig. 2:**
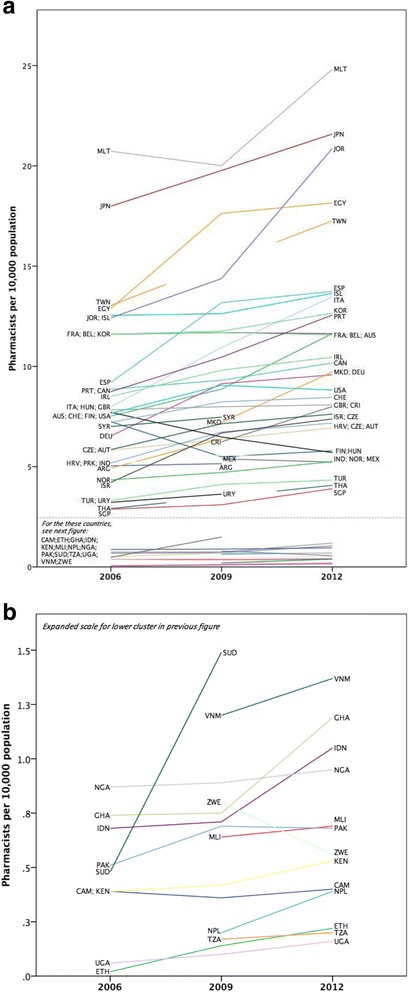
**a** Time trend for full data sample 2006–2012. **b** Time trend for low capacity data cases (data sub-set of **a**)

**Fig. 3:**
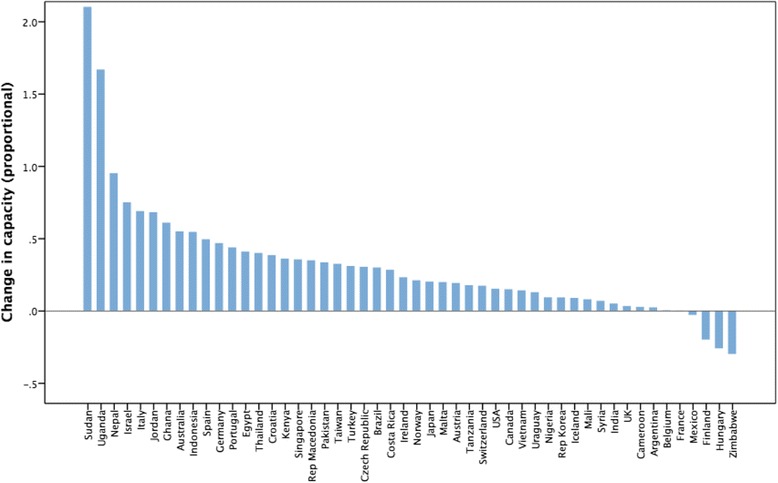
Relative capacity change as a Z-score (*N* = 50 country cases from 2006 to 2012)

Charting the change in workforce density over the period 2006–2012 provides a meaningful picture of workforce capacity trends. The full sample (*n* = 58 countries) is shown in Fig. 2a; countries and territories are identified by the ISO three-letter abbreviation (see Additional file 1 for alphabetic order) to maintain clarity. The cluster of countries at the bottom of Fig. 2a are those that have very low pharmacist densities and due to scaling of the vertical axis cannot easily be identified on this chart; Fig. 2b shows this cluster in expanded scale detail for comparison. Figure 3 shows relative changes (trends) in workforce capacity between 2006 and 2012 for individual countries with validated date points (data standardised using Z-scores for graphical scaling). As can be seen, for some countries, capacity has reduced during this time period.

**Fig. 4:**
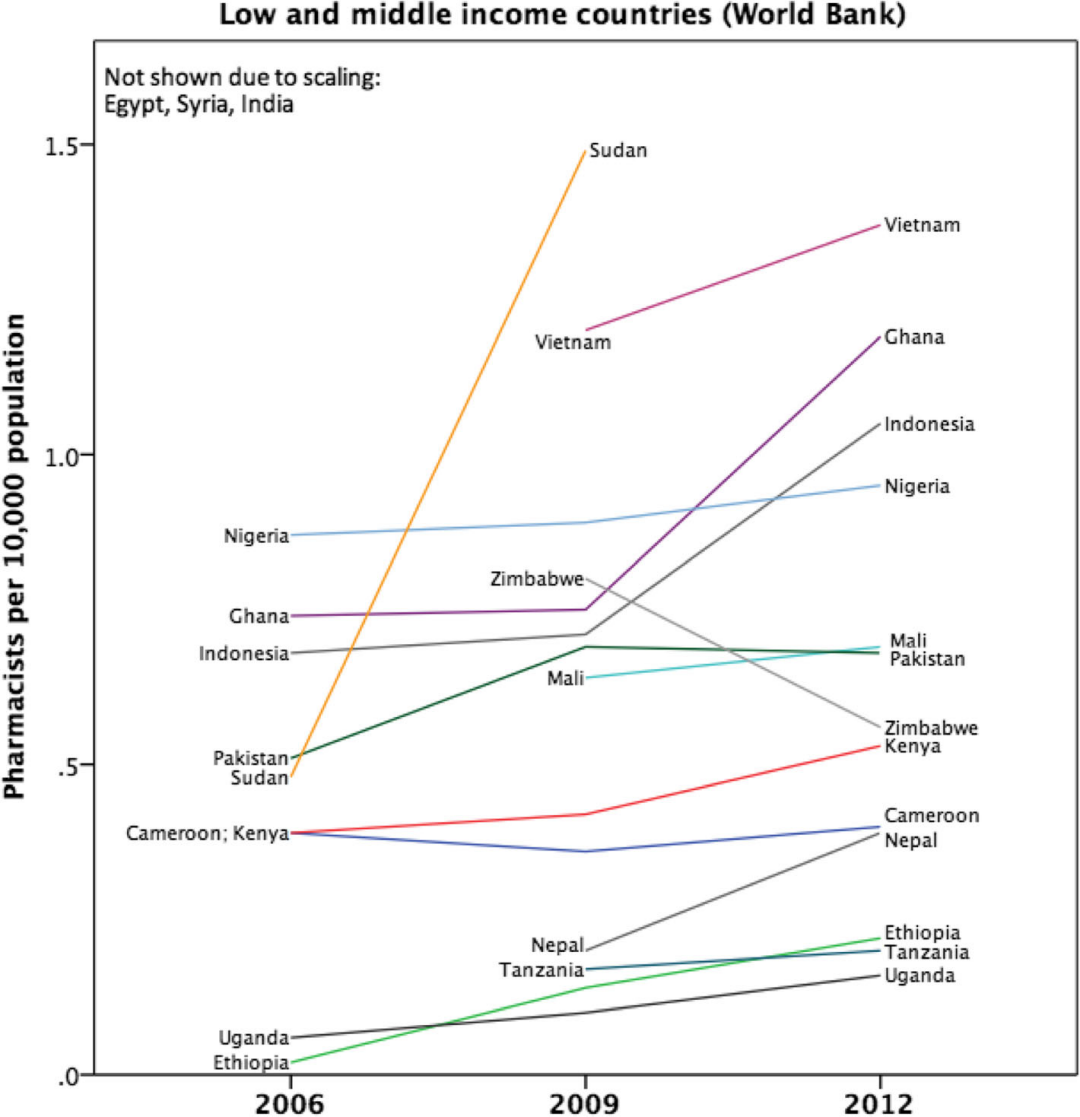
Time trend for low and lower middle income data cases 2006–2012

**Fig. 5:**
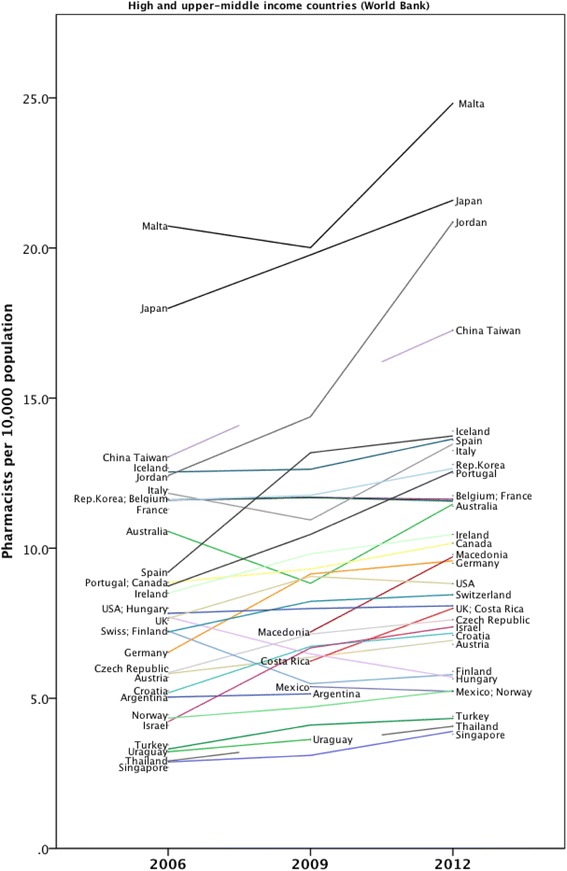
Time trend for high and upper middle income data cases 2006–2012

**Fig. 6:**
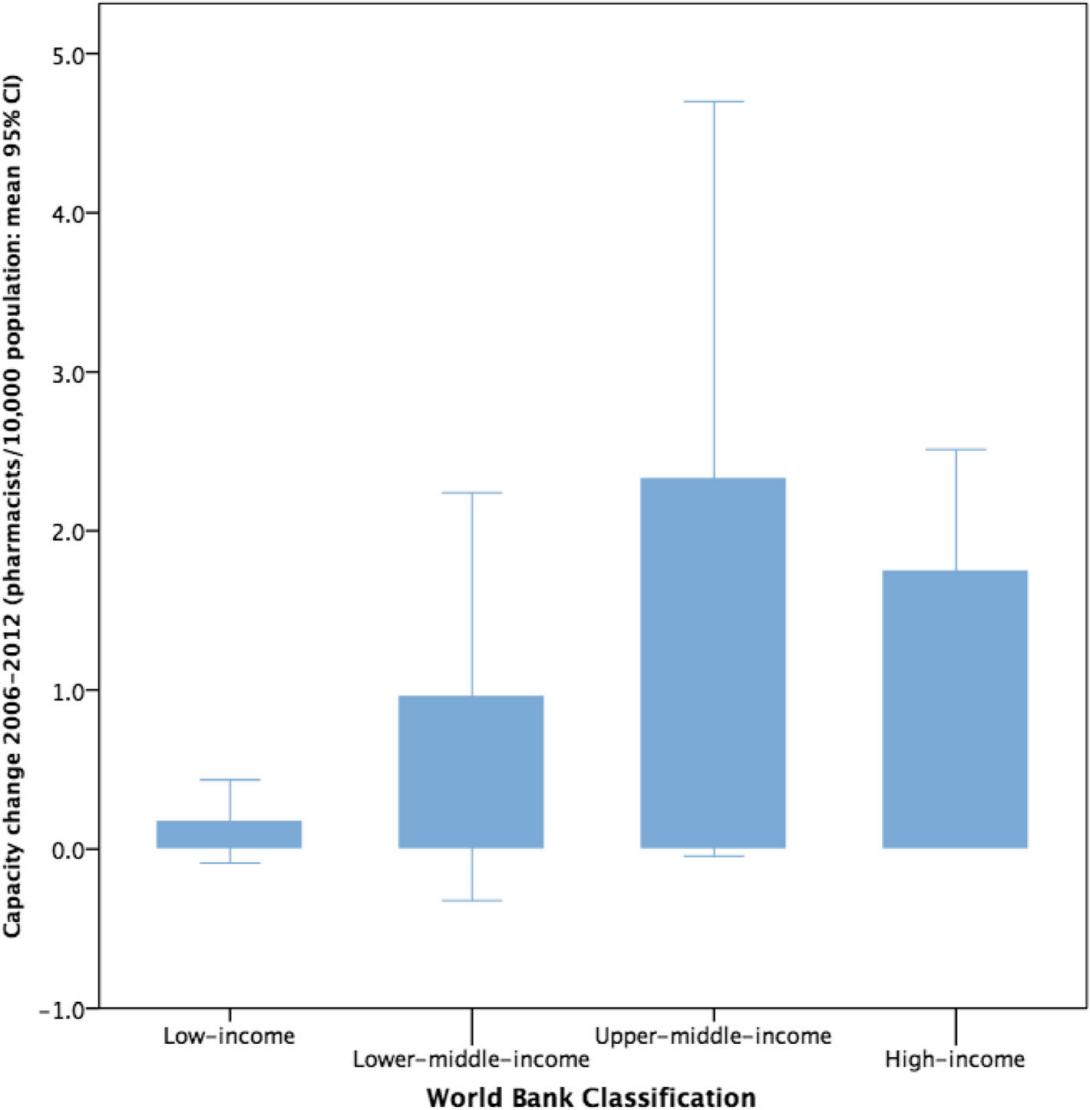
Capacity change (density) for World Bank-classified countries

The workforce density data was also examined by country-level income classification (World Bank) and presented as Figs. 4 and 5 for individual countries in the sample. Figure 6 shows the aggregated capacity change (as absolute density) across the time period using World Bank income level classification. Figure 6 also shows that an increased capacity trend is more prevalent in higher-income countries and is associated with economic factors as measured by World Bank income status. There is a significant association between absolute change in capacity (over time) and country-level higher-income status (Spearman’s rho = 0.419, *p* = 0.002). Although some African nations, for example, have made significant relative increases in capacity development (Uganda and Sudan have increased their own capacity by high percentages) in relation to other countries in absolute terms, Africa remains capacity-poor overall.

## Discussion

The countries and territories identified as having data for each of the time points 2006, 2009 and 2012 were present in all the WHO regions, with Europe having the most countries with data available and South East Asia the fewest. All WHO regions have experienced an increase in the density of pharmacists per 10 000 population over the period 2006–2012. Some decreases observed in density of pharmacists may be a result of net migration to other countries or other factors such as changes in national health policy. The negative effects on workforce supply of health worker migration and lack of supportive policy action, for example in providing quality educational infrastructure, has been documented [9].

Although the density of pharmacists has increased substantially in many lower-income countries such as Ethiopia, their baseline still remains low compared with those of higher-income countries. For both lower-income countries and higher-income countries, overall workforce numbers should be determined by strategic goals set by health policy-makers considering demand for health services. However, in reality reaching these goals may be challenging because of variations in the production of the workforce (e.g. numbers of schools of pharmacy and the lag time between educating and deploying staff) leaving lower-income countries with chronically low numbers in comparison with higher-income countries. Other influences that contribute to the dynamics of the workforce are the flows of pharmacists into and out of countries (immigration and emigration), part-time/interrupted practice and the proportion of workers reaching retirement age—all of these impact on the number of pharmacists available to nations.

We have shown changes in pharmacist capacity over the three time points of this study, displayed as an ‘absolute’ change in pharmacist density per capita*,* and additionally as relative *percent* change. It is important to clarify that percentage changes and absolute changes may tell different stories. A small change in the absolute pharmacist density in a country with very low capacity to begin with can result in a large percentage increase; conversely, a small percentage increase in a country with high density will result in a large absolute figure for workforce headcount. Within the WHO regions, there are also significantly wide variances that can make interpretation of aggregate statistics difficult. However, it is clear that the global trend for pharmacist workforce is increasing, and based on these data presented here, we estimate this increase to have been around 16% over the period concerned.

Again, converting changes to relative proportional changes may mask small absolute trends, particularly in smaller nations or nations with low capacity to begin with, with African nations having the smallest absolute changes in capacity. However, in this data set, not all countries have shown an increase in capacity, although changes in overall population denominators may contribute to the negative increases. The data also shows that some countries with small initial capacity (for example Sudan, Uganda) have a relative proportionally increased capacity, although once again their absolute capacity density remains very low in comparison with other, often more high-income, countries.

When considering changes in the density of pharmacists per country over time, it is useful to consider what the effect is on pharmacy workforce balance. When there is a national ‘gap’ between supply and demand for the pharmacy workforce, then imbalances occur. Variations in pharmacist density should not necessarily be considered a workforce imbalance. Changes may also reflect differences in role as pharmacists may be contributing to a nation’s healthcare in non-patient facing roles in the pharmaceutical industry and pharmaceutical manufacturing units. Additionally, density of pharmacists does not describe the productivity or distribution (and therefore accessibility, as generally healthcare workers tend to be more concentrated in urban rather than rural areas) of the workforce.

For instance, as health demand increases, the healthcare workforce needs to shift either by increasing its supply or by increases in its productivity. Absolute numbers of pharmacists do not reflect the issue of part-time workers, especially if their proportion has a greater increase relative to the growth in the number of pharmacists [8]. Failure to respond to increased health demand results in workforce imbalances and risks non-achievement of positive health outcomes.

Mapping pharmacists per capita with the World Bank classification gives an indication of the relationship of the workforce with economic indicators. Global Pharmacy Workforce Reports have shown a linear association with total pharmacist numbers and World Bank classification. In other words, the higher the level of a country’s income, the greater the number of pharmacists. Figure 6 illustrates that the capacity change over the period 2006–2012 (i.e. the change in the mean number of pharmacists per 10 000 population) showed the largest increase for the upper-middle-income countries compared with all other country income groups. This may reflect a greater increase in the growth in the economies classified as upper-middle income compared with other country income groups. Funding of the pharmacy workforce will also have an effect on its national workforce density.

Presenting data on workforce is always problematic, as has been alluded to earlier in this paper. For those countries where there were only two data points rather than three, the impact on workforce trends is less reliable. Likewise, data sourced from the web and literature searches may not be viewed as valid compared to that which was previously collated and validated from surveys. Presenting proportional change (which is easier to visualise) or absolute change (harder to interpret but more realistic) is challenging. Other limitations include the lack of direct association between capacity as a measure and whether there is a sufficient supply of pharmacists with the relevant competencies and skill mix. For countries with already low densities of pharmacists, it is possible that there is a gap between the need for pharmacy services and the actual supply of pharmacists.

## Conclusions

Although many WHO regions have experienced increases in their pharmacist workforce (notably the Eastern Mediterranean and Africa) over the period 2006–2012, countries and territories that are classified by the World Bank as low or lower middle income still have a low density of pharmacists compared with those nations classified as high or upper middle income [8]. It has been well documented that for low-income countries in particular there is a shortage of healthcare professionals needed to deal with the local disease burden. Such countries’ increases in workforce (where they can be achieved) might not be keeping pace with the increases in population and shifts in disease burden.

It is essential to monitor the global pharmacist workforce trends at regular time points so that decisions can be made as to how countries can deploy their workforce.

## Additional file

Additional file 1: ISO three-digit country abbreviations. (DOCX 25 kb)

## Abbreviations

FIP: International Pharmaceutical Federation
FIP*Ed*: FIP Education
GNI: Gross National Income
WDG: Workforce Development Goals
WHO: World Health Organization

## Footnotes

1 “Pharmacist” is defined in this paper as a licenced, regulated and/or registered health care professional, with accredited and regulated degree-entry initial education and training. We are not including data on pharmacy technicians, or pharmacy assistants, in this analysis.
