# Supplementary material for: An analysis of the global pharmacy workforce capacity trends from 2006 to 2012
Source: Hum Resour Health. 2018 Jan 11;16:3. doi: 10.1186/s12960-018-0267-y (PMC5765699; doi:10.1186/s12960-018-0267-y)
Supplement: Additional file 1: — ISO three-digit country abbreviations. (DOCX 25 kb) [file 12960_2018_267_MOESM1_ESM.docx]

| **Country** | **ISO 3-digit Country Code** | **Country** | **ISO 3-digit Country Code** |
| --- | --- | --- | --- |
| Australia | AUS | Israel | ISR |
| Austria | AUT | Italy | ITA |
| Belgium | BEL | Japan | JPN |
| Brazil | BRA | Jordan | JOR |
| Canada | CAN | Kenya | KEN |
| Croatia | HRV | Korea, Rep. | KOR |
| Czech Republic | CZE | Malta | MLT |
| Egypt, Arab Rep. | EGY | Nigeria | NGA |
| Ethiopia | ETH | Norway | NOR |
| Finland | FIN | Pakistan | PAK |
| France | FRA | Portugal | PRT |
| Germany | DEU | Singapore | SGP |
| Ghana | GHA | Spain | ESP |
| Hungary | HUN | Switzerland | CHE |
| Iceland | ISL | Turkey | TUR |
| India | IND | Uganda | UGA |
| Indonesia | IDN | United Kingdom | GBR |
| Ireland | IRL | United States | USA |
